# Respiratory Diseases of Small Ruminants

**DOI:** 10.1155/2014/373642

**Published:** 2014-12-30

**Authors:** Amit Kumar, Suresh K. Tikoo, Praveen Malik, Aruna T. Kumar

**Affiliations:** ^1^Department of Veterinary Microbiology, College of Veterinary Sciences, DUVASU, Mathura 281 001, India; ^2^Vaccinology & Immunotherapy Program, School of Public Health and VIDO-InterVac, University of Saskatchewan, 120 Veterinary Road, Saskatoon, SK, Canada S7N 5E3; ^3^Veterinary Type Culture Collection, National Research Centre on Equines, Hisar, Haryana 125 001, India; ^4^Directorate of Information and Publications of Agriculture, Kabi, New Delhi 110 012, India

Small ruminants are valuable assets for the Mediterranean, African, and Southeast Asian countries with the potential for providing meat, milk, and wool. These animals are highly susceptible to respiratory diseases, which account for almost 50% mortality amongst them. Irrespective of the etiology, the infectious respiratory diseases of sheep and goats contribute to 5.6 percent of the total diseases of small ruminants. The infectious respiratory disorders are classified into two groups: the diseases of upper respiratory tract including sinusitis caused by the larvae of parasites, nasal foreign bodies, gaseous irritation, and enzootic nasal tumors and the diseases of lower respiratory tract comprising mainly pneumonia. Often these are of infectious origin (bacterial, viral, or fungal). However, the role of the environmental pollutants, toxicants, and mechanical induction of respiratory distress may also be the cause of these abnormal conditions. Depending upon the environmental, physiological, and etiological factors, respiratory conditions might be acute, chronic, and/or progressive in nature.

To overcome such important disease conditions, information on their identification, prevention, cure, and control can improve the economic status and sustainability of holders of small ruminants. Thus an early, rapid, and specific diagnosis of such diseases holds great importance to reduce the losses. The advanced enzyme-linked immunosorbent assays (ELISAs) for the detection of antigens as well as antibodies directly from the samples are primarily available for all the disease conditions with specificity and sensitivity. Similarly, molecular diagnostic assays along with microsatellites comprehensively assist in diagnosis as well as treatment and epidemiological studies. The two reviews written by Chakraborty et al. and Kumar et al. discuss the advancements made in the diagnosis of common infectious respiratory diseases of sheep and goats, in general, and also the management of* Mycoplasma agalactiae*, a causal agent of classical contagious agalactia (CA). CA is a serious, economically important but neglected enzootic disease of small ruminants. Both reviews would certainly assist in designing appropriate prevention protocols and devising suitable control strategies to overcome such important respiratory diseases, thus alleviating the economic losses. The cross-reactivity and differential immunogenicity between* Mycoplasma agalactiae* and* Mycoplasma bovis*, two often confusing pathogens causing contagious agalactia, have also been elaborated in the research article authored by Kumar et al.

The research article by P. Scott details the antibiotic treatment response in sheep with chronic lung diseases where the diagnosis was based upon ultrasonographic examination of the lungs using 5 MHz linear and sector scanners with no consideration to the auscultation findings. The lung pathological examinations were considered a basis for comparison between sonographic findings and pathological changes.

Rahal et al. described in detail how a number of pathogenic microorganisms have been implicated in the development of respiratory diseases but the importance of environmental factors in the initiation and progress of the disease can never be overlooked. These environmental factors irritate the respiratory track producing stress in the microenvironment causing a decline in the immune status of the small ruminants and thereby assisting bacterial, viral, and parasitic infections in breaking down the tissue defense barriers. Environmental pollutants cause acute or chronic reactions as they deposit on the alveolar surface, which are characterized by inflammation or fibrosis and the exhibition of transitory or persistent tissue manifestation.

The disease development can be portrayed as three sets of two-way communications among pathogen, environment, and host but the interactions are highly variable. Moreover, the environmental scenario is never static; new compounds are introduced daily making a precise evaluation of the disease burden almost impossible. The last article in the issue presents a detailed overview of these interactions and the ultimate effect on the respiratory health of sheep and goat.

